# Editorial: A year in review: discussions in adrenal endocrinology

**DOI:** 10.3389/fendo.2023.1291582

**Published:** 2023-09-22

**Authors:** Ivana Kraljevic

**Affiliations:** ^1^ School of Medicine, University of Zagreb, Zagreb, Croatia; ^2^ Department of Endocrinology, University Hospital Centre Zagreb, Zagreb, Croatia

**Keywords:** adrenal gland, discussion, endocrinology, incidentaloma, pheochromocytoma, adrenal insufficiency, tumour microenvironment, partial adrenalectomy

## Introduction

The field of Adrenal Endocrinology has experienced remarkable growth over the last few years. As research and clinical practice in this domain continues to evolve, it becomes imperative to emphasize the critical role of informed discussions among researchers, clinicians, and stakeholders. In this editorial, we shed light on the significance of discussions in Adrenal Endocrinology. We present different topics and discuss various areas of Adrenal Endocrinology.

The article” *Predicting morphological and functional variations of benign adrenal incidentalomas in relation to initial characteristics*” discusses current issues for the follow-up of adrenal incidentalomas (Parazzoli et al.). According to the current guidelines of the European Society of Endocrinology (1), patients diagnosed with adrenal incidentalomas (AI) should undergo a comprehensive evaluation at the time of diagnosis to assess the potential malignancy of the tumor and the presence of excess adrenal hormones. If the adrenal mass is less than 4 cm and shows benign characteristics with normal hormone activity during the initial assessment, no further investigations are necessary due to the low risk of malignancy or functional changes (1).

However, some other guidelines (2) and consensus positions (3–5) recommend regular radiological and biochemical follow-up for all adrenal masses, regardless of their characteristics. The following is based on the possibility of changes in their nature over time, even if they appear benign initially.

Few studies have addressed the long-term follow-up of unresected AI, making the usefulness and timing of reassessment unclear. A recent study by Ceccato et al. (6) focused on radiological modifications (diameter and lipid content) in a large cohort of AI patients, according to their cortisol secretion, after a long-term follow-up. The authors suggest that follow-up imaging should be performed around 5 years after diagnosis, especially in patients with autonomous cortisol secretion (ACS), lipid-poor adenomas, and a large diameter at baseline.

Although there is no clear consensus on predictive criteria, adrenal adenomas larger than 2.4 cm may deserve more attention, as they pose a risk of functional progression. ACS may occur in a significant number of patients, especially when there is a larger initial adenoma diameter, higher cortisol levels, and cardiovascular risk factors. The occurrence of cortisol hypersecretion may exacerbate cardiovascular and metabolic comorbidities.

The risk of morphological and functional changes in AI increases over time, particularly after 5-10 years of follow-up, but it is currently difficult to predict. Therefore, discontinuing the follow-up in patients with NFAT may carry risks, and all these factors should be considered to determine the best management approach for these patients.

The article “*A Spatiotemporal Steroidogenic Regulatory Network in Human Fetal Adrenal Glands and Gonads*” presents an intriguing study with significant potential for advancing our understanding of steroid hormone regulation during human development (Wang et al.). In this research, the authors meticulously mapped the adrenal glands and gonads of fetuses aged 7-14 weeks. It sheds light on the role of fetal adrenal glands and gonads in influencing the process of sexual differentiation. The study’s results can be outlined as follows: The adrenal glands start expressing steroidogenic enzyme genes around seven weeks, producing steroid hormones much earlier than the testis. The expression patterns of steroidogenic enzyme genes in the testis suggest that it can synthesize testosterone *de novo* or utilize DHEA from the adrenal gland. Females exhibit an HSD3B2 expression peak at ten weeks. While the adrenal glands might synthesize small amounts of DHT, steroidogenic enzyme expression in ovaries remains limited until 14 weeks.

In the article “*Adrenal crisis in infants and young children with adrenal insufficiency: Management and prevention*” there are summarized current clinical practice standards for adrenal crisis and different treatment modalities in a group of children with adrenal insufficiency (Bizzari et al.).

Fifty-one children were investigated using various adrenal medications. The overall number of adrenal crisis episodes was 7.3/patient/yr in children <4 yrs and 4.9/patient/yr in children >4 yrs. Hospital admissions averaged 0.5/patient/yr in children <4 yrs and 0.53/patient/yr in children >4 yrs. The micronized weighted formulation showed promising results, with no suspected adrenal crises reported during the 6-month observation period.

Key measures to prevent adrenal crises include parental education on stress dosing (7), using parenteral hydrocortisone when needed, and communication devices to alert healthcare workers (8). Awareness of potential causes and promptly diagnosing AI in children with relevant symptoms are vital in managing adrenal crises.

The article “*The promising role of risk scoring system for Cushing syndrome: Time to reconsider current screening recommendations*” suggests that risk-scoring systems may offer significant potential (Lam-Chung and Cuevas-Ramos).

The authors review the latest progress in Cushing syndrome’s clinical risk scoring system. The prevalence of endogenous hypercortisolism is rising due to conditions like T2D, obesity, metabolic syndrome, and depression (9, 10). To address this, diagnostic scores based solely on clinical signs (11, 12) could be valuable for guiding physicians in conducting initial screening tests. A standardized approach using a clinical score system based on evidence can expedite the diagnosis of CS, leading to timely identification and reduced morbidity with fewer long-term consequences (13–15).

The article “*Total versus partial adrenalectomy in bilateral pheochromocytoma – a systematic review and meta-analysis*” addresses a significant and clinically relevant topic in managing bilateral pheochromocytoma (Zawadzka et al.).

The analysis comprised 25 studies involving 1444 patients. Patients who underwent partial adrenalectomy had a lower risk of losing adrenal hormone function during follow-up and needing steroid therapy (RR: 0.32, 95% CI: 0.26-0.38, P < 0.00001). They also had a lower odds ratio for developing acute adrenal crisis (OR: 0.3, 95% CI: 0.1-0.91, P=0.03). However, partial adrenalectomy was associated with a higher risk of local tumor recurrence than total adrenalectomy (OR: 3.72, 95% CI: 1.54-8.96, P=0.003) (16–26).

In five studies comparing TA and PA for pheochromocytoma (16, 17, 20–22), no significant difference in the development of metastases was found (OR 1.47, 95% CI: 0.48-4.44, P=0.5, I2 = 0%). The follow-up durations ranged from 4.9 to 12.2 years, with no metastases reported in either group during follow-up periods of 6 to 16.7 years.

In conclusion, partial adrenalectomy for bilateral pheochromocytoma offers a chance of preserving adrenal hormonal function but comes with an increased risk of local tumor recurrence. However, there was no significant difference in the risk of metastasis and overall mortality between the groups undergoing total or partial adrenalectomy.

The article “*Tumour microenvironment in pheochromocytoma and paraganglioma*” reviews these tumors’ microenvironment (TME) characteristics and provides valuable insights into their biology, behavior, and potential therapeutic targets (Martinelli et al.).

Pheochromocytomas and Paragangliomas (Pheo/PGL) are rare catecholamine-producing tumors with around 10-15% developing metastatic forms and a poor 37% mortality rate at five years (27, 28). SDHB mutations are associated with metastatic conditions (29). This review explores the roles of TME cells like cancer-associated fibroblasts and tumor-associated macrophages and non-cellular components like growth factors, extracellular vesicles, and extracellular matrix in Pheo/PGL growth and progression (30, 31). TME cells produce numerous growth factors and cytokines, facilitating close crosstalk with tumor cells. This interaction supports cancer cell survival, promotes angiogenesis, and fosters resistance to therapies (32, 33). Moreover, immune cells within the tumor release immunosuppressive mediators that dampen host-mediated antitumor responses, further aiding tumor progression (34). The significance of succinate as an oncometabolite and its receptor SUCNR1 in carcinogenesis is also analyzed (35, 36). Therefore, exploring novel molecular targets, including the TME, is crucial to enhance and diversify existing therapies for improved treatment outcomes.

## Conclusion

The significance of discussions in Adrenal Endocrinology cannot be overstated. By embracing open and informed dialogues, we can accelerate advancements in research, improve patient care, and address the challenges posed by adrenal gland disorders. Engaging in these discussions allows us to unlock the full potential of knowledge and collaboration in this field.

## Author contributions

IK: Writing – original draft.
